# Predicting cognitive resilience from midlife lifestyle and multi-modal MRI: A 30-year prospective cohort study

**DOI:** 10.1371/journal.pone.0211273

**Published:** 2019-02-19

**Authors:** Anya Topiwala, Sana Suri, Charlotte Allan, Vyara Valkanova, Nicola Filippini, Claire E. Sexton, Verena Heise, Enikő Zsoldos, Abda Mahmood, Archana Singh-Manoux, Clare E. Mackay, Mika Kivimäki, Klaus P. Ebmeier

**Affiliations:** 1 Department of Psychiatry, University of Oxford, Oxford, United Kingdom; 2 Wellcome Centre for Integrative Neuroimaging, Oxford, United Kingdom; 3 Centre for Research in Epidemiology and Population Health, INSERM, Villejuif, France; 4 Department of Epidemiology and Public Health, University College London, London, United Kingdom; Nathan S Kline Institute, UNITED STATES

## Abstract

**Background:**

There is significant heterogeneity in the clinical expression of structural brain abnormalities, including Alzheimer’s disease biomarkers. Some individuals preserve their memory despite the presence of risk factors or pathological brain changes, indicating resilience. We aimed to test whether resilient individuals could be distinguished from those who develop cognitive impairment, using sociodemographic variables and neuroimaging.

**Methods:**

We included 550 older adults participating in the Whitehall II study with longitudinal data, cognitive test results, and multi-modal MRI. Hippocampal atrophy was defined as Scheltens Scores >0. Resilient individuals (n = 184) were defined by high cognitive performance despite hippocampal atrophy (HA). Non-resilient participants (n = 133) were defined by low cognitive performance (≥1.5 standard deviations (S.D.) below the group mean) in the presence of HA. Dynamic and static exposures were evaluated for their ability to predict later resilience status using multivariable logistic regression. In a brain-wide analysis we tested for group differences in the integrity of white matter (structural connectivity) and resting-state networks (functional connectivity).

**Findings:**

Younger age (OR: 0.87, 95% CI: 0.83 to 0.92, p<0.001), higher premorbid FSIQ (OR: 1.06, 95% CI: 1.03 to 1.10, p<0.0001) and social class (OR 1 vs. 3: 4.99, 95% CI: 1.30 to 19.16, p = 0.02, OR 2 vs. 3: 8.43, 95% CI: 1.80 to 39.45, p = 0.007) were independently associated with resilience. Resilient individuals could be differentiated from non-resilient participants by higher fractional anisotropy (FA), and less association between anterior and posterior resting state networks. Higher FA had a significantly more positive effect on cognitive performance in participants with HA, compared to those without.

**Conclusions:**

Resilient individuals could be distinguished from those who developed impairments on the basis of sociodemographic characteristics, brain structural and functional connectivity, but not midlife lifestyles. There was a synergistic deleterious effect of hippocampal atrophy and poor white matter integrity on cognitive performance. Exploiting and supporting neural correlates of resilience could offer a fresh approach to postpone or avoid the appearance of clinical symptoms.

## Introduction

The term resilience is frequently used in a psychological context, referring to the ability to cope in the face of stressful life events. There is recent evidence that the concept of resilience is also meaningful and relevant for cognitive outcomes [1, 2]. Some individuals appear to maintain high memory function despite the presence of risk factors for impairment [3] or pathological brain changes, indicating resilience. For example, approximately one fifth of individuals positive for Alzheimer biomarkers have normal cognitive function, regardless of whether these are neuropathological findings on autopsy [4], amyloid on positron emission tomography (PET) imaging [5] or abnormal levels of cerebrospinal fluid (CSF) tau and amyloid [2, 6]. Similarly, the extent of functional deficits following stroke and traumatic brain injury can differ markedly [7, 8].

Two related concepts, brain reserve and cognitive reserve, have been cited as mechanisms to explain resilience, although the terms are often used interchangeably in the literature [9]. Reserve is proposed to moderate between brain measures and cognition. Brain reserve refers to differences in the anatomical properties of the brain, such as its size, which confer a passive buffer against the effects of damage [10, 11]. Cognitive reserve theory was initially borne out of epidemiological studies not examining the brain. Lower dementia risk was reported in those with certain socio-demographic characteristics, such as high education or intelligence, now often used as proxies for reserve [12, 13]. Cognitive reserve is proposed to reflect an active ability to optimize performance through the differential recruitment of brain networks, altered brain metabolism [14, 15], or alternative cognitive strategies. Functional brain imaging has revealed posterior-to-anterior or bilateral shifts in activation, and network de-differentiation in older adults, who successfully maintain their cognitive function [16, 17]. Impaired connectivity may predict cognitive impairment in Alzheimer’s disease [18]. Whether such strategies can compensate for structural brain adversity has not been investigated. Mounting evidence suggests that plasticity is possible even in the adult brain [19], making the distinction between passive brain reserve and active cognitive reserve models somewhat blurred and artificial [20]. Here we use the term resilience, which describes the observed phenomenon without mechanistic connotation.

Despite the vital implications for clinical care and research agenda, predictors and mechanisms underlying resilience have not been established [21]. Insights into cognitive resilience are likely to inform the interpretation and clinical significance of neuroimaging findings. Additionally, enhancing and supporting potential neural correlates of resilience may offer a fresh therapeutic approach, for example in Alzheimer’s disease, where current research is focusing with limited success on drugs to reduce the primary pathology [21–23]. There have been recent attempts to identify socio-demographic features of individuals resilient to high genetic Alzheimer’s disease risk [3, 24], beta amyloid and tau [25], and grey matter reductions [26, 27]. However, the importance of midlife lifestyle and clinical factors, and structural and functional brain connectivity has not yet been explored.

In our study, we defined and characterized resilience on the basis of cognitive performance in the presence of an established biomarker for Alzheimer’s disease (hippocampal atrophy, HA). Amongst those with hippocampal atrophy, we sought to identify socio-demographic, clinical and brain corollaries of good (“resilient”) versus poor (“non-resilient”) outcomes. Our first hypothesis, was that high social class and high full-scale IQ (FSIQ), often cited as markers of cognitive reserve [10], would be associated with higher resilience [12, 28]. Alternative cognitive strategies or differential network recruitment may facilitate maintained cognition. Hence our second hypothesis was that resilient individuals would have superior structural brain connectivity and distinct patterns of functional connectivity.

## Materials and methods

### Subjects and socio-demographic and lifestyle data

**Fig 1:**
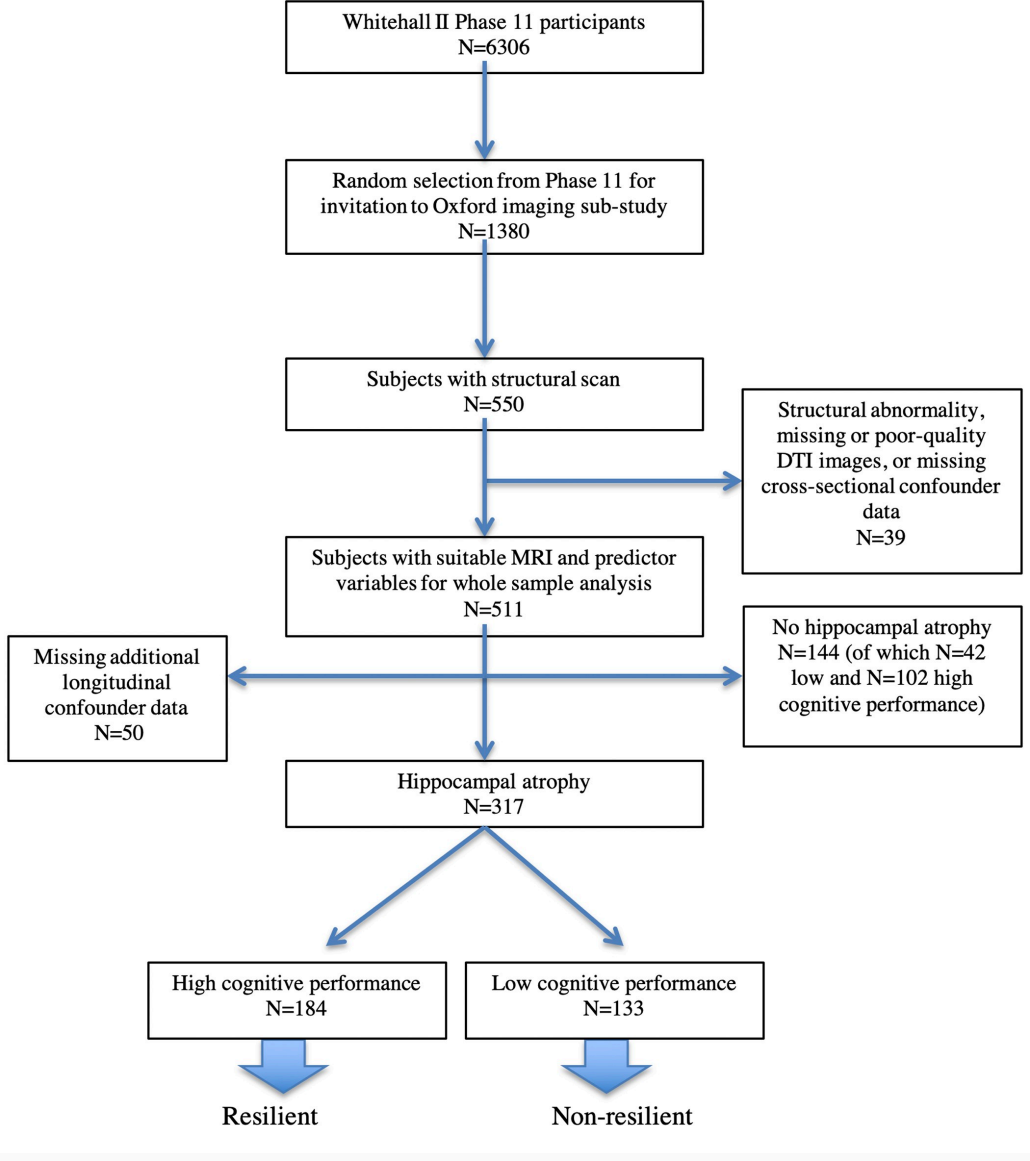
Selection of sample and definition of resilient and non-resilient groups. Hippocampal atrophy was defined using the Scheltens’ scale (>0) [32]. High cognitive performance was defined as <1.5 S.D below the mean on any cognitive test [33]. Low cognitive performance was defined as ≥1.5 S.D. below the mean on at least one cognitive test. **Abbreviations**–N–number, MRI–magnetic resonance imaging, DTI–diffusion tensor imaging.

Five hundred and fifty participants were randomly selected from the Whitehall II cohort for the imaging sub-study (2012–2016) [29]. Comparison to the larger sample is made in S1 Table. Apart from self-exclusion due to inability to attend the examination in Oxford and take part in an MRI study, post-hoc exclusions were due to incomplete or poor-quality images, gross structural abnormality, or missing cognitive or confounder data (Fig 1, S2 Table). Socio-demographic, health and lifestyle variables were measured prior to MRI over a follow-up period of approximately thirty years, in 1985–8 (Phase 1), 1991–3 (Phase 3), 1997–9 (Phase 5), 2003–4 (Phase 7), 2007–9 (Phase 9), and 2011 (Phase 11) (see S2 Table). Age, sex, active bilingualism, smoking, alcohol consumption, physical activity, depressive symptoms (General Health Questionnaire) and the Framingham Stroke Risk Score (FRS), were assessed by self-report questionnaire or clinical examination. Social class was determined according to occupation in 1991–3. Social networks were measured using questions derived by Berkman and Syme [30] and summarized on a scale. Current cognitive function and subjective memory complaints were assessed prior to the MRI scan with Montreal Cognitive Assessment (MoCA), Trail Making Test (TMT A and B), Rey-Osterrieth Complex Figure (RCF) copying, RCF immediate and delayed recall, Hopkins Verbal Learning Test (HVLT-R) total immediate (HVLT TR) and delayed (HVLT DR) recall, Digit Span (DSF/DSB/DSS) and Digit Coding (all from the Wechsler Adult Intelligent Scale-IV), lexical and semantic fluency, Boston Naming Test (BNT) and Test of Premorbid Function (TOPF). Short-term memory (recall of a 20-word list) was tested in: 1985–8, 1991–3, 1997–9, 2003–4, 2007–9, 2011 and 2015–6. FSIQ was estimated using the TOPF with sex and education adjustment [31]. History of Major Depressive Disorder was assessed prior to the MRI scan using the Structured Clinical Interview for DSM-IV.

Participants gave written informed consent to be approached for the study and gave separate written informed consent for every procedure included in the study, following a protocol and consent form approved by the respective Ethics of Research Committees. Capacity for consent was assessed by research workers trained in this procedure (EZ, AM). No formal psychological tests or assessments were used to determine whether participants were able to provide written informed consent. The ethics committee approved this procedure.

### Defining resilient and non-resilient participants

The resilient group of participants was defined by preserved cognitive function in the presence of HA (<1.5 S.D. below mean on all cognitive tests). HA was selected on the basis of its known association with cognitive impairment in the literature. It has been identified as one of the five most robust biomarkers for Alzheimer’s disease and is a supportive sign in the revised NINCDS-ADRDA diagnostic criteria [5, 34]. We also empirically tested its relation to cross-sectional and longitudinal cognition in our sample (see S3 & S4 Tables, S1 Fig). HA was identified using the well-validated and widely used semi-quantitative Scheltens visual rating scale as any atrophy score >0 [32, 35, 36] (S2 Fig). Although we have hippocampal volume measures, we elected to use a visual rating scale to identify hippocampal atrophy for a number of reasons: Visual ratings are highly sensitive and specific [32], in some cases more sensitive than volumetry [37]. They are clinically widely applicable, unlike volumetry, which necessitates specialist software and operator correction of segmentation. While volumetry assesses absolute, or at best relative volume (in relation to intracranial or grey matter volume), visual rating scales indicate anatomical abnormalities, such as visible and enlarged choroid fissures, enlarged lateral ventricles and hippocampal thickness. In other words, visual ratings account for grey matter, and inverse proportional changes in cerebrospinal fluid. Therefore, high scores may better reflect a pathological change, rather than smaller premorbid size. In contrast, volumetry (usually done after automatic segmentation of grey matter) only accounts for grey matter making such a distinction impossible. Inter- and intra-rater reliability is high (ICC = 0.8–0.9 and 0.7–0.9 respectively from our data) [28]. Reproducibility between quantitative approaches is not straightforward. Differences in operator judgement, scanner sequence, magnet strength and automated software can significantly impact results.

The (non-resilient) group of participants was defined by impaired cognitive performance in the presence of HA (defined as ≥1.5 S.D. below mean [33] in the absence of validated normative data for all tests in this age group) on *at least one* test. Whilst links between hippocampal volume and verbal memory are well described [38, 39], as also demonstrated in the predictive power of hippocampal atrophy for the trajectory of verbal memory in the Whitehall II study (see S4 Table and S1 Fig) we chose additionally to verbal memory to consider other cognitive domains following empirical analyses demonstrating relationships between hippocampal volume and performance on multiple tests (see S3 and S5 Tables, S1 Text). In the absence of collateral and concurrent medical information about health and functional ability, we were unable to make a diagnosis of Mild Cognitive Impairment or dementia. However, the absence of a reported clinical diagnosis, ranges of impairment, participants’ ability to give informed consent and to make their way to Oxford from across the country independently, suggest that there was nobody with a probable diagnosis of dementia.

### MRI analysis

All MRI scans were acquired between 2012 and 2015 at the University of Oxford Wellcome Centre for Integrative Neuroimaging (WIN), using a 3 Tesla Siemens Verio scanner. T1-weighted MPRAGE, fluid-attenuated inversion recovery (FLAIR), 60-direction diffusion tensor (DTI) and multi-band EPI 3T MRI sequences were used. T1-weighted images were processed using FSL tools (www.fmrib.ox.ac.uk/fsl) [40] and ‘fsl_anat (Beta version)’ (http://fsl.fmrib.ox.ac.uk/fsl/fslwiki/fsl_anat).

#### Hippocampal metrics

Two hippocampal metrics were examined. First, the presence of hippocampal atrophy, used to identify resilient and non-resilient subjects, was assessed using the Scheltens visual rating scale. Scores >0 were used as an indication of atrophy [41]. Three clinicians, blind to measured volumes and participant characteristics independently rated each subject. Discrepancies were resolved following a consensus meeting. Second, hippocampal volumes were automatically extracted using FIRST [42], corrected for intracranial volume (ICV, by dividing by total intracranial volume*100, as is standard in the literature) and averaged across sides. Volume measures were only used for comparison (see S4 Fig), and when exploring accessory hypotheses of associations between cognitive performance measures and hippocampal volume.

#### Voxel-based morphometry analysis

To discount the possibility that the non-resilient subjects had a more advanced disease process (indicated by grey matter differences) than the resilient subjects, group differences in brain-wide grey matter density were examined. Voxel-based morphometry (VBM) is an objective method to compare grey matter density between groups in each voxel (smallest distinguishable image volume) of the structural image.

To avoid overfitting, confounders were included in each model if they were: 1) an established risk factor for the outcome measure, 2) not thought to lie on the causal chain between exposure and outcome. Premorbid FSIQ and social class were included to isolate brain differences independent of cognitive reserve. Adjustment was made in the VBM analysis for: age, sex, FRS, alcohol consumption, social class and FSIQ.

#### Diffusion tensor imaging analysis

Diffusion tensor images indicate the directional preference of water diffusion in neural tissue and allow inferences about the structural integrity of white matter tracts. Images were corrected for head movement and eddy currents and brain masks generated using BET. Fractional anisotropy (FA), mean (MD), axial (AD) and radial diffusivity (RD) maps were generated using DTIFit (http://fsl.fmrib.ox.ac.uk/fsl/fdt). Tract-Based Spatial Statistics (TBSS) were used [43] to perform voxelwise statistical analysis. Pre-processing prepared images for registration to standard space. Mean and skeletonized FA, MD, RD and AD images were created and thresholded. Lastly each FA, MD, RD and AD image was projected onto the relevant skeleton. To detect group differences between resilient and non-resilient subjects, a generalised linear model (GLM) was applied using permutation-based non-parametric testing (randomise) [44], correcting for multiple comparisons across space (threshold-free cluster enhancement, TFCE). Additionally for subsequent analysis, masks of the corpus callosum body were created using the ICBM-DTI-81 white-matter labels atlas [45] and used to extract mean FA indices. Adjustment for confounding included: age, sex, FRS, FSIQ, social class, alcohol consumption and depressive symptoms.

#### Resting state functional connectivity analysis

We used a hypothesis-free method to identify group differences in the synchronicity of responses across the brain between functionally distinct resting state network nodes [46]. Participants were scanned on a 3T Siemens Magnetom Verio (Erlangen, Germany) scanner with a 32-channel head coil, at the FMRIB Center, Oxford. T1-weighted structural MRI (multi-echo MPRAGE sequence with motion correction) and multiband echo-planar imaging rs-fMRI scans (voxel ¼ 2 mm isotropic, TR ¼ 1.3 s, acquisition time ¼ 10 min 10 s, multi-slice acceleration factor ¼ 6, number of volumes ¼ 460) were acquired. Rs-fMRI data were pre-processed (motion correction, brain extraction, high-pass temporal filtering at 100s, field-map correction) using FSL tools. MELODIC pre-processing includes motion correction, brain extraction, high-pass temporal filtering (cut-off 150 seconds) and field map correction [40]. To reduce noisy components, a data-cleaning approach was used. Following single-subject independent components analysis (ICA), FMRIB’s ICA-based X-noisefier (FIX) was used to automatically classify and regress out artefactual components [47]. FIX was “trained” on hand-classified ICA components on a matched training set (Whitehall II MB6). Data were registered affine to structural images using FLIRT [48]. FNIRT was used to register images into standard (MNI) space [49]. High dimensionality (dimensions = 100) ICA was performed on the pre-processed images to produce a study-specific template of spatial maps [50]. This template was used to extract time series using the dual regression approach [51]. Time courses were fed into FSLNets (v0.6) to perform network modeling [46]. Nodes were classified as ‘good’ (n = 58) or ‘bad’ (n = 42) (white matter, physiological noise, MRI or movement artifacts) [52]. The netmat was created and partial correlations were calculated [46]. Matlab was used to reorder the nodes after a hierarchical clustering of the group-average correlation netmat (S3 Fig*)*. A nodes x edges matrix was created. Group differences were examined with randomise, controlling for multiple comparisons (family-wise error), age, sex, FSIQ and social class.

### Other statistical analyses

All analyses outlined below were done with R version 3.4.0 [53]. Descriptive data were summarized for all subjects, and separately by group, according to variable type and distribution (Table 1).

**Table 1:** Sample characteristics.

	Mean (S.D.)^1^, median (IQR)^2^ or N (%)^3^			Comparison of resilient and non-resilient groups
	Whole sample (N = 511)	Resilient (N = 184)	Non-resilient (N = 133)	
Age at scan, **years**	69.5 (5.3)^1^	69.1 (4.9)^1^	72.7 (5.5)^1^	3.7 (2.5 to 4.9), p<0.0001^4^
Female, **N (%)**	101 (19.8)^3^	30 (16.3)^3^	21 (15.8)^3^	0.015, -0.09 to 0.1, p = 0.9^6^
Education, **years**	14 (5)^2^	16 (6)^2^	13 (6)^2^	9931.5, p = 0.004^5^
FSIQ^8^	118.0 (10.3)^1^	122.3 (7.9)^1^	116.2 (10.7)^1^	-5.7 (-7.9 to -3.6), p<0.0001^4^
Social class, **N (%)**^9^				
4	4 (0.8)	0 (0)	0 (0)	14.8, p = 0.01^7^
3	35 (6.8)	4 (2.2)	15 (11.3)	
2	392 (76.7)	140 (76.1)	102 (76.6)	
1	80 (15.7)^3^	40 (21.7)^3^	16 (12.0)^3^	
Systolic blood pressure, **mmHg**	120.6 (13.6)^1^	120.6 (13.9)^1^	121.8 (12.2)^1^	1.4 (-1.8 to 4.5), p = 0.4^4^
Diastolic blood pressure, **mmHg**	75.6 (9.6)^1^	75.5 (9.8) ^1^	76.7 (8.9) ^1^	0.8 (-1.3 to 3.0), p = 0.4^4^
History of Major Depressive disorder, **N (%)**^**10**^	90 (19.6)^3^	54 (29.3)^3^	30 (22.6)^3^	3.8, (0.002 to 0.2), p = 0.05^6^
General Health Questionnaire, anxiety score	3.9 (2.6) ^1^	3.6 (2.3) ^1^	3.7 (2.9) ^1^	0.1 (-0.5 to 0.7), p = 0.7^4^
Alcohol, **weekly units**	8 (12)^2^	10 (13)^2^	8 (13)^2^	11489, p = 0.4^5^
Moderate exercise, **weekly N**	2.3 (0.9) ^1^	2.1 (0.8) ^1^	2.2 (0.9) ^1^	0.01 (-0.2 to 0.2), p = 0.9^4^
Club attendance, weekly **N**^11^	2.9 (1.2) ^1^	2.8 (1.2) ^1^	2.9 (1.2) ^1^	0.06 (-0.2 to 0.4), p = 0.7^4^

^1^ Mean (standard deviation).

^2^ Median (interquartile range).

^3^ Number (percentage).

^4^ Mean difference (95% confidence interval), p value.

^5^W statistic, p value.

^6^ Odds ratio (95% confidence intervals), p value.

^7^ Pearson statistic, p value.

^8^Estimated from Test of Premorbid Function with education and sex adjustment.

^9^Social class based on occupation, Phase 3: 4 = skilled manual, 3 = skilled non-manual, 2 = managerial, 1 = professional.

^10^Structured Clinical Interview for DSM IV (SCID) at time of MRI scan.

^11^At time of MRI scan.

Baseline (unless otherwise stated) characteristics of whole sample with DTI and adequate confounder information, and resilient and non-resilient subgroups.

**Abbreviations:** N–number, S.D.–standard deviation, IQR–interquartile range, FSIQ–full-scale intelligence quotient, DTI–diffusion tensor imaging.

Logistic regression was used to identify significant predictors of resilience status (binary outcome: ‘resilient’ versus ‘non-resilient’, see above). Social class and FSIQ were added to the model as predictors. Education was not included due to concerns about multicollinearity with the concomitant inclusion of premorbid FSIQ (which was more closely associated with resilience (see Table 1), was education adjusted and suffered fewer ceiling effects). Longitudinal data on putative confounding factors were initially included in the model based on knowledge of the literature: age, sex, exercise (mean weekly hours of moderate activity over phases), Framingham Risk Score (mean 10 year stroke risk (%) over phases), alcohol (mean units per week over phases), smoker (proportion of phases), depressive symptoms (mean GHQ score over phases), and social networks (mean score over phases). Non-significant predictors (p>0.05) were excluded from the final model presented.

**Fig 2:**
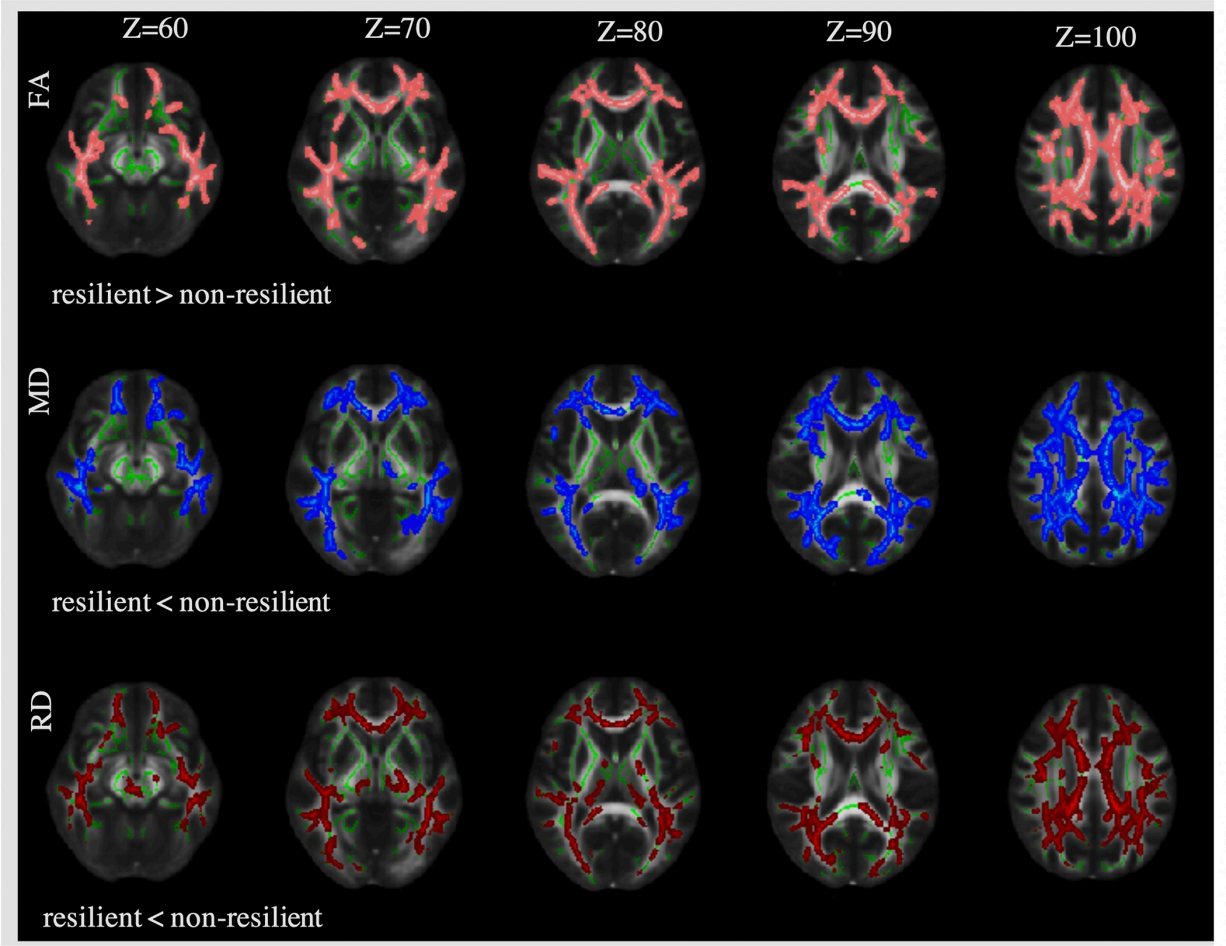
Significant group differences in white matter integrity. Voxels where there were significant differences in white matter integrity between resilient (N = 184) and non-resilient (N = 133) groups are shown in colour. Three indices of white matter integrity are shown: fractional anisotropy differences are shown in pink, mean diffusivity differences in blue, and radial diffusivity differences in red. Images were generated from the Tract-Based Spatial Statistics analyses and thresholded at p<0.05. The mean FA skeleton is shown in green. There were no significant differences in axial diffusivity differences (not shown). Analyses were adjusted for: age, sex, premorbid FSIQ, social class, Framingham Risk Score, alcohol consumption, depressive symptoms (TFCE). **Abbreviations:** Z–spatial axis, FA–fractional anisotropy, MD–mean diffusivity, RD–radial diffusivity, FSIQ–full-scale intelligence quotient, TFCE–threshold-free cluster enhancement.

In order to test whether MRI-based predictors of resilience identified from preceding analyses were associated specifically with resilience rather than just better verbal memory, we used multiple linear regression on the whole sample with DTI (with and without HA) (n = 511). To reduce multiple testing, we used HVLT DR as measure for verbal memory. This was on the basis of a strong established association with the hippocampus in the literature [54] and in our empirical analysis (S3 Table). We hypothesized that a true MRI predictor of resilience would in the presence of hippocampal atrophy differ in its ability to predict memory function (dependent variable) from its predictive power in participants without hippocampal atrophy. We tested this adding the interaction term: predictor*hippocampal volume to the independent variables. In the interests of a parsimonious analysis, we chose a single MRI measure to reflect ‘white matter integrity’, i.e. FA of the body of the corpus callosum. First, significant group differences existed in this region as shown by the TBSS analysis (Fig 2), and second, as the corpus callosum is a large tract separate from grey matter, it increases confidence that the FA measurement reflects only white matter. Models were adjusted for age, sex, Framingham Risk Score, alcohol consumption, depressive symptoms, premorbid FSIQ and social class. All hypothesis tests were two-sided and statistical significance was deemed as p<0.05.

## Results

### Identification of resilient subjects

The MRI Substudy sample was comparable to the whole Whitehall II Phase 11 cohort from which it had been selected randomly (S1 Table). A relative excess of women took part in the imaging study. Overall mean years of education and depressive symptoms were higher, BP measures lower in the subsample. Availability of lifetime risk data across previous phases of the sub-sample was satisfactory (S2 Table). A summary of participant characteristics is given in Table 1. The distribution of Scheltens hippocampal atrophy scores is illustrated in S2 Fig. Of 527 participants in the MRI Study, 317 showed Scheltens scores > 0 suggesting a degree of hippocampal atrophy. Of these, we identified 184 individuals with the resilience phenotype and 133 who were non-resilient participants. Mean intracranial volume-adjusted volumes were associated with atrophy scores, but this association was by no means perfect (S4 Fig). Seventy-two of those we defined as non-resilient reported subjective complaint about their cognition at the time of scanning, as opposed to twenty-eight in the resilient group. Empirical justification for our resilience definition is included in S1 Text: There was no significant group difference in hippocampal volume (as expected based on the selection methods), nor large disparities in grey matter density (S5 Fig). Therefore, it is unlikely that our resilience phenotype was an artefact of a less advanced disease process with less wide-spread volumetric changes. A small group (n = 42) of subjects had impairment on cognitive testing in the absence of small hippocampi, implying extra-hippocampal changes, thus not fitting our definitions of resilient or non-resilient participants, and therefore outside of our formulated research question.

### Sociodemographic correlates of resilience

Higher premorbid FSIQ (OR per 1-point increase: 1.06, 95% CI: 1.03 to 1.10, p<0.0001) and social class (OR for 1 vs. 3: 4.99, 95% CI: 1.30 to 19.16, p = 0.02, OR for 2 vs. 3: 8.43, 95% CI: 1.80 to 39.45, p = 0.007) independently predicted resilience (after controlling for age and sex; Table 2). These odds were not confounded by exercise habits, social networks, vascular risk (Framingham Risk Score), depressive symptoms, bilingualism, alcohol consumption or smoking, measured repeatedly over the 30 years, which also did not independently predict resilience.

**Table 2:** Sociodemographic correlates of resilience.

	N	Unadjusted odds ratio (95% CI)	Adjusted^3^ odds ratio (95% CI)
Age, years		0.88 (0.84 to 0.92), p<0.001	0.87 (0.83 to 0.92), p<0.001
Male Sex	266		
Female Sex	51	1.04 (0.57 to 1.91), p = 0.90	2.22 (0.98 to 5.01), p = 0.06
FSIQ^1^		1.07 (1.04 to 1.10), p<0.001	1.06 (1.03 to 1.10), p<0.001
Social class^2^			
3	19		
2	242	9.38 (2.70 to 32.59), p<0.001	8.43 (1.80 to 39.45), p = 0.007
1	56	5.15 (1.66 to 16.00), p = 0.005	4.99 (1.30 to 19.16), p = 0.02

^1^ Premorbid FSIQ (education and sex adjusted) according to Test of Premorbid Function performed at the time of scan.

^2^ Social class based on occupation at Phase 3: 1 = professional, 2 = managerial, 3 = skilled non-manual. No subjects included in this subset analysis were in social class 4 (manual).

^3^ For all other predictors in model lists in column one.

Results from logistic regression analyses, predicting odds of resilience (visually rated hippocampal atrophy *and* absent cognitive impairment, n = 184)) vs. non-resilient participants (visually rated hippocampal atrophy *and* cognitive impairment on ≥1 test, n = 133).

**Abbreviations:** CI–confidence interval, p–p value, FSIQ–full-scale intelligence quotient.

### Structural connectivity in resilient and non-resilient participants

**Fig 3:**
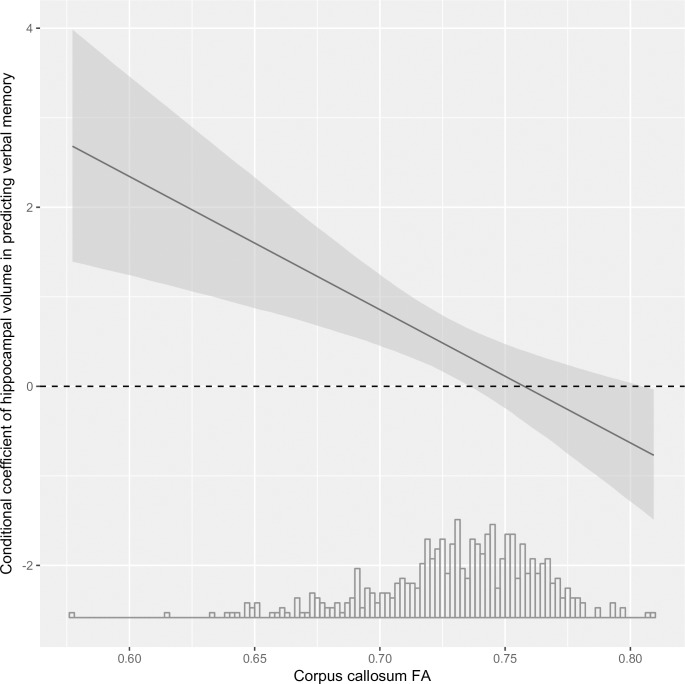
White matter integrity becomes an increasingly more important correlate of verbal memory, as hippocampal volume gets smaller. Dependence of estimated conditional regression coefficients for corpus callosum fractional anisotropy (independent variable) in predicting HVLT DR (dependent variable) on hippocampal volume (% intracranial volume). Grey shading represents 95% confidence intervals. Values are on the basis of a regression model including all subjects (N = 511) which included the following confounders: age, sex, Framingham Risk Score, depressive symptoms, alcohol consumption, social class and premorbid FSIQ. **Abbreviations**: HVLT DR–Hopkins Verbal Learning Test Delayed Recall, ICV–intracranial volume, FSIQ–full-scale intelligence quotient.

In the brain-wide TBSS analyses, resilient individuals showed increased fractional anisotropy (FA), and reduced mean diffusivity (MD) and radial diffusivity (RD) in multiple tracts compared with non-resilient individuals, but no differences in axial diffusivity (AD; Fig 2). Furthermore, in the separate regression analyses of the entire MRI sample irrespective of hippocampal atrophy (n = 511), there was a significant interaction (β -14.9.08, 95% CI: -23.21 to -6.64, P = 0.0004) between corpus callosum FA and hippocampal volume in predicting verbal memory (Fig 3). Strikingly, in subjects with large hippocampi (i.e. >2.25% ICV), corpus callosum FA did not predict cognitive performance. Amongst those with smaller hippocampi, FA became increasingly important (S1 Fig). In contrast, interaction terms for hippocampal volume and social class or FSIQ were not significant.

### Functional connectivity in resilient and non-resilient participants

**Fig 4:**
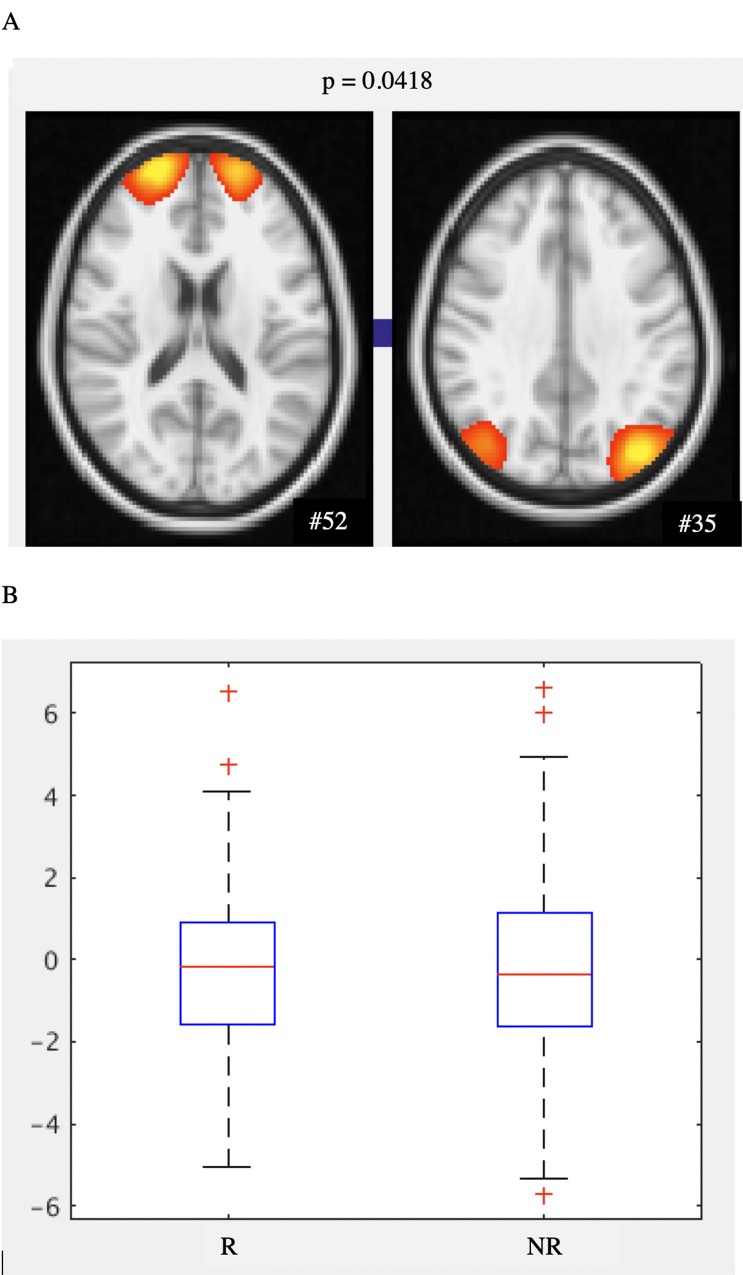
Functional connectivity differences between resilient and non-resilient participants. A) Resting state (negative) functional connectivity between network nodes #52 (central executive network, left image) and #35 (default mode network, right image). The p-value indicates significance of group difference in connectivity between these nodes. B) Boxplot showing partial correlation (edge strength) between nodes 35 and 52 in resilient (R) and non-resilient (NR) groups.

The functional connectivity between nodes #52 and #35 was weaker in the resilient group compared to the non-resilient group (Fig 4). One node (#52) of the pair was in frontal pole of the central executive network (CEN), and the other (#35) in the inferior parietal lobe (IPL) of the default mode network [55]. The population average connectivity matrix (S3 Fig) indicates that for the entire sample, the functional association between nodes #52 and #35 was weakly negative. However, there is not necessarily a relationship between the strength (or even sign) of the population average connection between two nodes and the extent to which inter-subject variability in the connection strength correlates with a given behavioural measure [46].

## Discussion

We combined multimodal neuroimaging and functional cognitive data to define a phenotype of resilience. Sociodemographic factors, but not lifestyle, were associated with resilience. Resilient subjects’ brains were characterized by higher structural integrity of white matter tracts and weaker inter-network functional connections.

### Comparison with other studies

As predicted, high premorbid FSIQ and social class (in addition to age) were independently associated with resilience, in the presence of HA [28]. However, it appears these effects can be explained by shared variance with cognitive test performance (general intelligence or ‘G’ factor), rather than a synergistic compensatory effect. In the Whitehall II sample at least, such factors, often referred to as indices of cognitive reserve, reflect higher baseline memory performance rather than greater compensatory capacity [10]. Our finding is in agreement with previous Whitehall II findings in dementia [56]. It also raises concerns that links between cognitive reserve indices and **cognitive impairment** could result from detection bias, if only cross-sectional cognitive performance is examined

White matter microstructure appears to partially mitigate the effect of HA on verbal memory in our sample, suggesting a potential ‘synergistic’ deleterious effect of hippocampal and white matter damage on cognitive performance. It is not clear whether resilient participants in our study have avoided age-related decline in white matter (reduced “brain battering”) and have greater capacity to tolerate damage (brain reserve), or whether differences are due to plasticity. Compensatory changes, including increased number of axons or increased myelination are possible, as have been reported in response to brain lesions [57].

Our functional connectivity results appear to challenge the general notion that “*stronger is better*”. Most resting-state fMRI studies to date have only described intra-network connections, often studying large brain networks as a whole. Our approach allowed us to examine both intra- and inter-network connectivity, which has been of greater interest in recent studies. While we observed no group differences in intra-network connections, participants in the resilient group appeared to have weaker functional association (in the form of an inverse partial correlation) between the CEN and DMN compared with non-resilient subjects. Functional brain networks in the elderly typically become less distinct due to an *increase* in inter-network strength of connections along with a relative *decrease* in intra-network connections in older compared to younger adults [58]. Here, a relatively weaker inter-network connection between the CEN and default mode nodes was associated with the resilience phenotype, suggesting that this group may maintain more distinct network structure in older age.

The CEN has been linked to demanding externally orientated tasks [59] and the DMN, mediates internally directed self-referential processes [60]. There are several reports that anti-correlation between the CEN and default mode networks is important for brain function [61, 62] and correlates with higher cognitive performance [63]. However, others have described a u-shaped relationship with aging, with compensatory increases in segregation of networks linked to reductions in structural integrity [64]. Our results appear to be more consistent with the latter, however without longitudinal imaging, it is impossible to determine whether the functional differences observed here are a compensation for pathological changes within the brain (HA) or represent lifelong differences between resilient and non-resilient groups. We therefore interpret the functional connectivity results with caution.

### Strengths and limitations

We consider three advances of our work over existing studies. First, the depth and duration of the phenotyping allowed a thorough long-term examination of lifestyle contributions to resilience, largely ignored by previous cross-sectional studies [25, 27]. Second, the breadth of the cognitive tests and measurement of longitudinal decline have not been considered in published work [25]. Third, use of multi-modal MRI and voxel-based brain analyses allowed us to examine the relationship between resilience, specifically defined, and structural and functional brain connectivity.

In spite of these strengths, some limitations need to be highlighted. The brain correlates of resilience we identified were in the context of analyses examining potential moderators of the effect of hippocampal atrophy on cognition. Other brain/cognition relationships may have entirely different moderating variables.

We used cross-sectional hippocampal atrophy ratings, so cannot exclude the possibility, of unlikely face validity, that these reflect lifelong differences rather than age-related or even pathological change. Whilst longitudinal imaging could be helpful, scan intervals of more than a couple of years add further confounders, because of developments in the physical scanner and imaging sequences. Links between hippocampal volume and current and future cognition are well established [38], and replicated in our sample. Whilst HA is a diagnostic marker for Alzheimer’s disease [34], we do not wish to claim subjects in our sample had specific markers of Alzheimer’s pathology in the absence of knowledge of tau or amyloid status. HA has also been reported in depression, neurotoxic injuries, other causes of dementia, and in moderate drinkers [41]. On the other hand, hippocampal sparing has been described in atypical Alzheimer’s disease [65] so we may have systematically excluded some participants with early Alzheimer’s pathology but large hippocampi. However, as our primary interest was in exploring tolerance to hippocampal atrophy, irrespective of its etiology, we do not think this detracts from the findings. We used a clinical visual rating scale to group subjects on the basis of HA, as there are no established ‘normal’ vs. ‘abnormal’ cut offs for *volumetric* data. The Scheltens scale has been widely used and is well validated, is applicable to clinical settings, and aligns to some extent with volumetric measures in our sample [32, 35, 36].

Standard deviation cut-offs rather than normative values were used to categorize subjects on cross-sectional cognitive performance. Our cut-offs do not necessarily generalize to other samples or indicate pathological deficits. Many of the tests we used do not have robust normative data. Even for the better investigated MoCA, there is no fine-grained breakdown for effects of age or education, and little data on older subjects relevant to our sample. There is continuing debate about what constitutes the ‘best’ normative comparison group for neurocognitive tests, and studies vary on the stringency with which they exclude cognitively impaired subjects. As with any observational study, we cannot deny the possibility that some individuals we defined as ‘resilient’, will develop cognitive impairment after our study period (right censoring).

We are uncertain about the generalizability of our findings, as the Whitehall II cohort is not representative of the wider population. By example, it has a low proportion of women, reflective of the 1980s UK civil service. Lastly, we cannot exclude a selection bias. Those most cognitively impaired are not unlikely to have been included in the study, which required travel to Oxford and a neuropsychological assessment. Although this may have attenuated some of the associations, it is an unlikely source of major bias.

### Implications of findings

This study provides one avenue of investigation into how brain findings may translate into future clinical symptoms. We envisage several potential clinical and research implications of our study. First, clinical interpretation of neuroimaging findings must take account of sociodemographic factors. Second, future research in cognitive impairment and dementia should not ignore multiple synergistic pathological contributions. Finally, a fresh approach to delaying symptoms in those with pathological changes may be exploiting and supporting neural correlates of resilience, such as white matter microstructure. To date, therapeutic research has been focusing on agents to reduce a single primary pathology, with limited success [22]. Exercise [66], vascular risk [67] and brain stimulation with electrical or magnetic currents have been associated with white matter integrity [68], and even neuroplasticity. Fruitful leads may therefore include tight control of cardiovascular risk factors with antihypertensives and lipid-lowering drugs, physical training programs or even brain stimulation. The approach may be particularly suitable in individuals whose scans have revealed early indicators of structural damage but exhibit minimum functional deterioration. Even small increases in resilience may postpone or avoid onset of symptoms in a clinically significant fashion.

## Supporting information

S1 Table Comparison of the imaging sample with the whole Whitehall II cohort at Phase 11. The MRI sample consisted of all those in the Oxford imaging sub-study with at least a structural MRI image (but not necessarily diffusion tensor imaging) and cross-sectional sociodemographic and lifestyle data.
(PDF)

S2 Table Completeness of lifestyle and health data at each study phase. (PDF)

S3 Table Cross-sectional cognitive performance and hippocampal volume. P-values result from hypothesis tests (likelihood tests) comparing regression models with and without hippocampal volume. N = 511. Models were adjusted for: age, sex, FSIQ and social class.
(PDF)

S4 Table Longitudinal verbal memory decline and hippocampal atrophy status. Estimates are from mixed effects models (binomial regression) fitted longitudinal memory test scores over time (study Phase 11 to 12). Models were adjusted for: age, sex, FSIQ, social class, time from study baseline and hippocampal atrophy status. Estimates represent the odds of memory recall. The ‘Time x hippocampal atrophy’ interaction term tests whether there was a significant difference in memory decline over time between subjects with and without hippocampal atrophy.
(PDF)

S5 Table Cross-sectional cognitive test data for resilient and non-resilient groups. (PDF)

S1 Fig Modelling longitudinal change in memory test scores according to hippocampal size for a typical subject. Predictions are made on the basis of mixed effects models (using N = 511 subjects) with cognitive testing (memory recall/20 words) performed before MRI at study Phase 11 and after the MRI in Phase 12, for a mean age of 75 years (at scan) and premorbid IQ of 118, according to cross-sectional hippocampal atrophy. Hippocampal atrophy is defined on the basis of the Scheltens scale (score>0).
(PDF)

S2 Fig Distribution of Scheltens ratings for hippocampal atrophy on whole imaged sample. Counts indicate number of participants from the whole imaged sample with hippocampal atrophy (left (L) and right (R) sides) as rated by the semi-quantitative Scheltens scale. N = 511.
(PDF)

S3 Fig Functional connectome consisting of hierarchical ordering of the 58 functionally distinct brain regions as network nodes, and the connectivities between these as network edges. The strengths of the estimated network edges are elements in a N_nodes_ x N_nodes_ network matrix. Boxes above the diagonal signify full correlations (thought to represent direct and indirect connections) and those below the diagonal represent partial correlations (used to infer only direct connections between nodes). Based on data from 317 subjects.
(PDF)

S4 Fig Relationship between Scheltens ratings of hippocampal atrophy and volumetric hippocampal measures. Violin plots (outline includes all data points; red diamond indicates mean) display Scheltens visual rating scores against automated hippocampal (as a % of total intracranial volume) for left and right sides. Red diamonds indicate mean hippocampal volumes. N = 511.
(PDF)

S5 Fig Grey matter in resilient and non-resilient subjects. Voxel-based morphometry analysis results. Orange/red blobs represent voxels where there is significantly higher grey matter density in resilient (N = 184) compared to cognitively impaired (N = 133) groups. Colour bar indicates 1-p values. Analysis was adjusted for: age, sex, alcohol consumption, Framingham Risk Score, FSIQ, social class and TFCE-corrected.
(PDF)

S1 Text Empirical justification for choice of resilience metrics. (PDF)

## References

[1] Mok VC, Lam BY, Wong A, Ko H, Markus HS, Wong LK. Early-onset and delayed-onset poststroke dementia [mdash] revisiting the mechanisms. Nature Reviews Neurology. 2017. 10.1038/nrneurol.2017.16 28211452

[2] Livingston G. Dementia prevention, intervention, and care. Lancet. 2017;S0140-6736(17):31363–6. 10.1016/S0140-6736(17)31363-6 28735855

[3] Kaup AR, Nettiksimmons J, Harris TB, Sink KM, Satterfield S, Metti AL, et al Cognitive resilience to apolipoprotein E ε4: contributing factors in black and white older adults. JAMA neurology. 2015;72(3):340–8. 10.1001/jamaneurol.2014.3978 25599330 PMC4624320

[4] Price JL, McKeel DW, Buckles VD, Roe CM, Xiong C, Grundman M, et al Neuropathology of nondemented aging: presumptive evidence for preclinical Alzheimer disease. Neurobiology of aging. 2009;30(7):1026–36. 10.1016/j.neurobiolaging.2009.04.002 19376612 PMC2737680

[5] Jack CR, Knopman DS, Jagust WJ, Petersen RC, Weiner MW, Aisen PS, et al Tracking pathophysiological processes in Alzheimer's disease: an updated hypothetical model of dynamic biomarkers. The Lancet Neurology. 2013;12(2):207–16. 10.1016/S1474-4422(12)70291-0 23332364 PMC3622225

[6] Andreasen N, Minthon L, Clarberg A, Davidsson P, Gottfries J, Vanmechelen E, et al Sensitivity, specificity, and stability of CSF-tau in AD in a community-based patient sample. Neurology. 1999;53(7):1488–. 10534256 10.1212/wnl.53.7.1488

[7] Umarova RM. Adapting the concepts of brain and cognitive reserve to post-stroke cognitive deficits: Implications for understanding neglect. Cortex. 2016. 10.1016/j.cortex.2016.12.006 28049565

[8] Schneider EB, Sur S, Raymont V, Duckworth J, Kowalski RG, Efron DT, et al Functional recovery after moderate/severe traumatic brain injury A role for cognitive reserve? Neurology. 2014;82(18):1636–42. 10.1212/WNL.0000000000000379 24759845 PMC4211893

[9] Okonkwo OC, Vemuri P. Stemming the Alzheimer tsunami: introduction to the special issue on reserve and resilience in Alzheimer’s disease. Brain imaging and behavior. 2017;11(2):301–3. 10.1007/s11682-017-9677-z 28116651 PMC5409879

[10] Stern Y. Cognitive reserve in ageing and Alzheimer's disease. The Lancet Neurology. 2012;11(11):1006–12. 10.1016/S1474-4422(12)70191-6 23079557 PMC3507991

[11] Harrison TM, Maass A, Baker SL, Jagust WJ. Brain morphology, cognition, and β-amyloid in older adults with superior memory performance. Neurobiology of aging. 2018;67:162–70. 10.1016/j.neurobiolaging.2018.03.024 29665578 PMC5955827

[12] Stern Y, Gurland B, Tatemichi TK, Tang MX, Wilder D, Mayeux R. Influence of education and occupation on the incidence of Alzheimer's disease. Jama. 1994;271(13):1004–10. 8139057

[13] Wang H-X, MacDonald SW, Dekhtyar S, Fratiglioni L. Association of lifelong exposure to cognitive reserve-enhancing factors with dementia risk: a community-based cohort study. PLoS medicine. 2017;14(3):e1002251 10.1371/journal.pmed.1002251 28291786 PMC5349652

[14] Morbelli S, Perneczky R, Drzezga A, Frisoni GB, Caroli A, Van Berckel BN, et al Metabolic networks underlying cognitive reserve in prodromal Alzheimer disease: a European Alzheimer disease consortium project. Journal of Nuclear Medicine. 2013;54(6):894–902. 10.2967/jnumed.112.113928 23591639

[15] Bastin C, Yakushev I, Bahri MA, Fellgiebel A, Eustache F, Landeau B, et al Cognitive reserve impacts on inter-individual variability in resting-state cerebral metabolism in normal aging. Neuroimage. 2012;63(2):713–22. 10.1016/j.neuroimage.2012.06.074 22796505

[16] Cabeza R. Hemispheric asymmetry reduction in older adults: the HAROLD model. Psychology and aging. 2002;17(1):85 11931290 10.1037//0882-7974.17.1.85

[17] Davis SW, Dennis NA, Daselaar SM, Fleck MS, Cabeza R. Que PASA? The posterior–anterior shift in aging. Cerebral cortex. 2008;18(5):1201–9. 10.1093/cercor/bhm155 17925295 PMC2760260

[18] Franzmeier N, Düzel E, Jessen F, Buerger K, Levin J, Duering M, et al Left frontal hub connectivity delays cognitive impairment in autosomal-dominant and sporadic Alzheimer’s disease. Brain. 2018;141(4):1186–200. 10.1093/brain/awy008 29462334 PMC5888938

[19] Zatorre RJ, Fields RD, Johansen-Berg H. Plasticity in gray and white: neuroimaging changes in brain structure during learning. Nature neuroscience. 2012;15(4):528–36. 10.1038/nn.3045 22426254 PMC3660656

[20] Barulli D, Stern Y. Efficiency, capacity, compensation, maintenance, plasticity: emerging concepts in cognitive reserve. Trends in cognitive sciences. 2013;17(10):502–9. 10.1016/j.tics.2013.08.012 24018144 PMC3840716

[21] Bennett DA. Lack of Benefit With Idalopirdine for Alzheimer Disease: Another Therapeutic Failure in a Complex Disease Process. Jama. 2018;319(2):123–5. 10.1001/jama.2017.19700 29318261

[22] Doody RS, Raman R, Farlow M, Iwatsubo T, Vellas B, Joffe S, et al A phase 3 trial of semagacestat for treatment of Alzheimer's disease. New England Journal of Medicine. 2013;369(4):341–50. 10.1056/NEJMoa1210951 23883379

[23] Jobke B, McBride T, Nevin L, Peiperl L, Ross A, Stone C, et al Setbacks in Alzheimer research demand new strategies, not surrender. Public Library of Science; 2018. 10.1371/journal.pmed.1002518 PMC5828351 29486005

[24] McDermott KL. Sex effects in predictors of memory resilience for carriers of Alzheimer’s genetic risk: University of Alberta; 2016.

[25] Rentz DM, Mormino EC, Papp KV, Betensky RA, Sperling RA, Johnson KA. Cognitive resilience in clinical and preclinical Alzheimer’s disease: the Association of Amyloid and Tau Burden on cognitive performance. Brain imaging and behavior. 2017;11(2):383–90. 10.1007/s11682-016-9640-4 27738998 PMC5391311

[26] van Loenhoud AC, Wink AM, Groot C, Verfaillie SC, Twisk J, Barkhof F, et al A neuroimaging approach to capture cognitive reserve: Application to Alzheimer's disease. Human Brain Mapping. 2017. 10.1002/hbm.23695 PMC6867139 28631336

[27] Groot C, van Loenhoud AC, Barkhof F, van Berckel BN, Koene T, Teunissen CC, et al Differential effects of cognitive reserve and brain reserve on cognition in Alzheimer disease. Neurology. 2018;90(2):e149–e56. 10.1212/WNL.0000000000004802 29237798

[28] Topiwala A, Allan CL, Valkanova V, Zsoldos E, Filippini N, Sexton CE, et al Resilience and MRI correlates of cognitive impairment in community-dwelling elders. The British Journal of Psychiatry. 2015:bjp. bp. 114.152363. 10.1192/bjp.bp.114.152363 PMC4629074 26338988

[29] Filippini N, Zsoldos E, Haapakoski R, Sexton CE, Mahmood A, Allan CL, et al Study protocol: the Whitehall II imaging sub-study. BMC psychiatry. 2014;14(1):159. 24885374 10.1186/1471-244X-14-159 PMC4048583

[30] Berkman LF, Syme SL. Social networks, host resistance, and mortality: a nine-year follow-up study of Alameda County residents. American journal of Epidemiology. 1979;109(2):186–204. 425958 10.1093/oxfordjournals.aje.a112674

[31] Wechsler D. Test of premorbid functioning. UK version (TOPF UK). UK: Pearson Corporation 2011.

[32] Scheltens P, Leys D, Barkhof F, Huglo D, Weinstein H, Vermersch P, et al Atrophy of medial temporal lobes on MRI in" probable" Alzheimer's disease and normal ageing: diagnostic value and neuropsychological correlates. Journal of Neurology, Neurosurgery & Psychiatry. 1992;55(10):967–72. 10.1136/jnnp.55.10.967 PMC1015202 1431963

[33] Petersen RC, Doody R, Kurz A, Mohs RC, Morris JC, Rabins PV, et al Current concepts in mild cognitive impairment. Archives of neurology. 2001;58(12):1985–92. 11735772 10.1001/archneur.58.12.1985

[34] McKhann GM, Knopman DS, Chertkow H, Hyman BT, Jack CR, Kawas CH, et al The diagnosis of dementia due to Alzheimer’s disease: Recommendations from the National Institute on Aging-Alzheimer’s Association workgroups on diagnostic guidelines for Alzheimer's disease. Alzheimer's & dementia. 2011;7(3):263–9. 10.1016/j.jalz.2011.03.005 PMC3312024 21514250

[35] Harper L, Fumagalli GG, Barkhof F, Scheltens P, O’Brien JT, Bouwman F, et al MRI visual rating scales in the diagnosis of dementia: evaluation in 184 post-mortem confirmed cases. Brain. 2016:aww005. 10.1093/brain/aww005 PMC4806219 26936938

[36] Korf ES, Wahlund L-O, Visser PJ, Scheltens P. Medial temporal lobe atrophy on MRI predicts dementia in patients with mild cognitive impairment. Neurology. 2004;63(1):94–100. 15249617 10.1212/01.wnl.0000133114.92694.93

[37] Wahlund L-O, Julin P, Lindqvist J, Scheltens P. Visual assessment of medial temporal lobe atrophy in demented and healthy control subjects: correlation with volumetry. Psychiatry Research: Neuroimaging. 1999;90(3):193–9. 10466738 10.1016/s0925-4927(99)00016-5

[38] Raz N, Gunning-Dixon FM, Head D, Dupuis JH, Acker JD. Neuroanatomical correlates of cognitive aging: evidence from structural magnetic resonance imaging. Neuropsychology. 1998;12(1):95 9460738 10.1037//0894-4105.12.1.95

[39] Golomb J, Kluger A, de Leon MJ, Ferris SH, Convit A, Mittelman MS, et al Hippocampal formation size in normal human aging: a correlate of delayed secondary memory performance. Learning & Memory. 1994;1(1):45–54. 10467585

[40] Smith SM, Jenkinson M, Woolrich MW, Beckmann CF, Behrens TE, Johansen-Berg H, et al Advances in functional and structural MR image analysis and implementation as FSL. Neuroimage. 2004;23:S208–S19. 10.1016/j.neuroimage.2004.07.051 15501092

[41] Topiwala A, Allan CL, Valkanova V, Zsoldos E, Filippini N, Sexton C, et al Moderate alcohol consumption as risk factor for adverse brain outcomes and cognitive decline: longitudinal cohort study. bmj. 2017;357:j2353 10.1136/bmj.j2353 28588063 PMC5460586

[42] Patenaude B, Smith SM, Kennedy DN, Jenkinson M. A Bayesian model of shape and appearance for subcortical brain segmentation. Neuroimage. 2011;56(3):907–22. 10.1016/j.neuroimage.2011.02.046 21352927 PMC3417233

[43] Smith SM, Jenkinson M, Johansen-Berg H, Rueckert D, Nichols TE, Mackay CE, et al Tract-based spatial statistics: voxelwise analysis of multi-subject diffusion data. Neuroimage. 2006;31(4):1487–505. 10.1016/j.neuroimage.2006.02.024 16624579

[44] Winkler AM, Ridgway GR, Webster MA, Smith SM, Nichols TE. Permutation inference for the general linear model. Neuroimage. 2014;92:381–97. 10.1016/j.neuroimage.2014.01.060 24530839 PMC4010955

[45] Crain B, Mori S. MRI atlas of human white matter. Amsterdam, Elsevier; 2005.

[46] Smith SM, Vidaurre D, Beckmann CF, Glasser MF, Jenkinson M, Miller KL, et al Functional connectomics from resting-state fMRI. Trends in cognitive sciences. 2013;17(12):666–82. 10.1016/j.tics.2013.09.016 24238796 PMC4004765

[47] Griffanti L, Salimi-Khorshidi G, Beckmann CF, Auerbach EJ, Douaud G, Sexton CE, et al ICA-based artefact removal and accelerated fMRI acquisition for improved resting state network imaging. Neuroimage. 2014;95:232–47. 10.1016/j.neuroimage.2014.03.034 24657355 PMC4154346

[48] Jenkinson M, Smith S. A global optimisation method for robust affine registration of brain images. Medical image analysis. 2001;5(2):143–56. 11516708 10.1016/s1361-8415(01)00036-6

[49] Andersson JL, Jenkinson M, Smith S. Non-linear registration, aka Spatial normalisation FMRIB technical report TR07JA2. FMRIB Analysis Group of the University of Oxford 2007;2.

[50] Smith SM. The future of FMRI connectivity. Neuroimage. 2012;62(2):1257–66. 10.1016/j.neuroimage.2012.01.022 22248579

[51] Beckmann CF, Mackay CE, Filippini N, Smith SM. Group comparison of resting-state FMRI data using multi-subject ICA and dual regression. Neuroimage. 2009;47(Suppl 1):S148.

[52] Suri S, Topiwala A, Filippini N, Zsoldos E, Mahmood A, Sexton CE, et al Distinct resting-state functional connections associated with episodic and visuospatial memory in older adults. NeuroImage. 2017. 10.1016/j.neuroimage.2017.07.049 PMC5678287 28756237

[53] Team RC. R: A Language and Environment for Statistical Computing. 2015.

[54] Sass KJ, Spencer D, Kim J, Westerveld M, Novelly R, Lencz T. Verbal memory impairment correlates with hippocampal pyramidal cell density. Neurology. 1990;40(11):1694–. 2234424 10.1212/wnl.40.11.1694

[55] Smith SM, Fox PT, Miller KL, Glahn DC, Fox PM, Mackay CE, et al Correspondence of the brain's functional architecture during activation and rest. Proceedings of the National Academy of Sciences. 2009;106(31):13040–5. 10.1073/pnas.0905267106 PMC2722273 19620724

[56] Rusmaully J, Dugravot A, Moatti J-P, Marmot MG, Elbaz A, Kivimaki M, et al Contribution of cognitive performance and cognitive decline to associations between socioeconomic factors and dementia: A cohort study. PLoS medicine. 2017;14(6):e1002334 10.1371/journal.pmed.1002334 28650972 PMC5484463

[57] Head D, Buckner RL, Shimony JS, Williams LE, Akbudak E, Conturo TE, et al Differential vulnerability of anterior white matter in nondemented aging with minimal acceleration in dementia of the Alzheimer type: evidence from diffusion tensor imaging. Cerebral Cortex. 2004;14(4):410–23. 15028645 10.1093/cercor/bhh003

[58] Geerligs L, Renken RJ, Saliasi E, Maurits NM, Lorist MM. A brain-wide study of age-related changes in functional connectivity. Cerebral cortex. 2015;25(7):1987–99. 10.1093/cercor/bhu012 24532319

[59] Seeley WW, Menon V, Schatzberg AF, Keller J, Glover GH, Kenna H, et al Dissociable intrinsic connectivity networks for salience processing and executive control. Journal of Neuroscience. 2007;27(9):2349–56. 10.1523/JNEUROSCI.5587-06.2007 17329432 PMC2680293

[60] Raichle ME, MacLeod AM, Snyder AZ, Powers WJ, Gusnard DA, Shulman GL. A default mode of brain function. Proceedings of the National Academy of Sciences. 2001;98(2):676–82. 10.1073/pnas.98.2.676 PMC14647 11209064

[61] Fox MD, Snyder AZ, Vincent JL, Corbetta M, Van Essen DC, Raichle ME. The human brain is intrinsically organized into dynamic, anticorrelated functional networks. Proceedings of the National Academy of Sciences of the United States of America. 2005;102(27):9673–8. 10.1073/pnas.0504136102 15976020 PMC1157105

[62] Chan MY, Park DC, Savalia NK, Petersen SE, Wig GS. Decreased segregation of brain systems across the healthy adult lifespan. Proceedings of the National Academy of Sciences. 2014;111(46):E4997–E5006. 10.1073/pnas.1415122111 PMC4246293 25368199

[63] Ng KK, Lo JC, Lim JK, Chee MW, Zhou J. Reduced functional segregation between the default mode network and the executive control network in healthy older adults: A longitudinal study. Neuroimage. 2016;133:321–30. 10.1016/j.neuroimage.2016.03.029 27001500

[64] Marstaller L, Williams M, Rich A, Savage G, Burianová H. Aging and large-scale functional networks: white matter integrity, gray matter volume, and functional connectivity in the resting state. Neuroscience. 2015;290:369–78. 10.1016/j.neuroscience.2015.01.049 25644420

[65] Murray ME, Graff-Radford NR, Ross OA, Petersen RC, Duara R, Dickson DW. Neuropathologically defined subtypes of Alzheimer's disease with distinct clinical characteristics: a retrospective study. The Lancet Neurology. 2011;10(9):785–96. 10.1016/S1474-4422(11)70156-9 21802369 PMC3175379

[66] Voss MW, Heo S, Prakash RS, Erickson KI, Alves H, Chaddock L, et al The influence of aerobic fitness on cerebral white matter integrity and cognitive function in older adults: Results of a one‐year exercise intervention. Human brain mapping. 2013;34(11):2972–85. 10.1002/hbm.22119 22674729 PMC4096122

[67] Maillard P, Seshadri S, Beiser A, Himali JJ, Au R, Fletcher E, et al Effects of systolic blood pressure on white-matter integrity in young adults in the Framingham Heart Study: a cross-sectional study. The Lancet Neurology. 2012;11(12):1039–47. 10.1016/S1474-4422(12)70241-7 23122892 PMC3510663

[68] Lyden H, Espinoza R, Pirnia T, Clark K, Joshi S, Leaver A, et al Electroconvulsive therapy mediates neuroplasticity of white matter microstructure in major depression. Translational psychiatry. 2014;4(4):e380. 24713861 10.1038/tp.2014.21 PMC4012285

