# Supplementary material for: Predicting cognitive resilience from midlife lifestyle and multi-modal MRI: A 30-year prospective cohort study
Source: PLoS One. 2019 Feb 19;14(2):e0211273. doi: 10.1371/journal.pone.0211273 (PMC6380585; doi:10.1371/journal.pone.0211273)
Supplement: S1 Table — The MRI sample consisted of all those in the Oxford imaging sub-study with at least a structural MRI image (but not necessarily diffusion tensor imaging) and cross-sectional sociodemographic and lifestyle data. (PDF) [file pone.0211273.s001.pdf]

S1 Table: Comparison of the imaging sample with the whole Whitehall II cohort at Phase 11

	MRI Sample N=527			Phase 11 Participants N=6306			Difference in means or proportions (95% CI)
	N	Mean/%	S.D.	N	Mean/%	S.D.	
Age, years	527	69.6	5.3	6306	69.8	5.9	-0.2 (-0.7 to 0.3), p=0.5
Sex<br>Female<br>Male	527<br>103<br>424	 19.5%<br>80.5%		6306<br>1947<br>4459	 29.3%<br>70.7%		-0.1 (-0.2 to -0.1), p<0.001
Full time education, years	527	14.6	3.2	5101	15.1	4.2	-0.5 (-0.9 to -0.1), p=0.008
CES-D scale	527	5.0	5.8	5855	7.3	7.6	-2.3 (-3.0 to -1.6), p<0.001
Systolic BP, mmHg	527	140.8	17.6	5652	127.8	16.5	13.0 (11.5 to 14.5), p<0.001
Diastolic BP, mmHg	527	76.5	10.6	5652	70.8	9.9	5.7 (4.8 to 6.6), p<0.001

The MRI sample consisted of all those in the Oxford imaging sub-study with at least a structural MRI image (but not necessarily diffusion tensor imaging) and cross-sectional sociodemographic and lifestyle data.

Abbreviations: MRI – magnetic resonance imaging, N – number, CI – confidence intervals, S.D. – standard deviation, CES-D Center for Epidemiological Studies Depression Scale, BP – blood pressure.
