# Supplementary material for: Predicting cognitive resilience from midlife lifestyle and multi-modal MRI: A 30-year prospective cohort study
Source: PLoS One. 2019 Feb 19;14(2):e0211273. doi: 10.1371/journal.pone.0211273 (PMC6380585; doi:10.1371/journal.pone.0211273)
Supplement: S2 Table — (PDF) [file pone.0211273.s002.pdf]

S2 Table: Completeness of lifestyle and health data at each study phase.

	Cases with missing data, n (total sample = 511)					
Study phase	*General Health Questionnaire depression score*	*Alcohol units weekly*	*Moderate exercise, weekly hours*	*Smoking status*	*Framingham Stroke Risk Score*	*Social network scale*
1	4	1	8	1	*	3
3	1	32	51	32	16	37[1]
5	32	15	32	2	28	24
7	18	1	1	13	22	12
9	9	6	2	1	9	*
11	7	0	2	2	0	*
Oxford	*	4	0	0	0	*

*Not assessed
[1]assessed at Phase 2
