# Supplementary material for: Predicting cognitive resilience from midlife lifestyle and multi-modal MRI: A 30-year prospective cohort study
Source: PLoS One. 2019 Feb 19;14(2):e0211273. doi: 10.1371/journal.pone.0211273 (PMC6380585; doi:10.1371/journal.pone.0211273)
Supplement: S3 Table — P-values result from hypothesis tests (likelihood tests) comparing regression models with and without hippocampal volume. N = 511. Models were adjusted for: age, sex, FSIQ and social class. (PDF) [file pone.0211273.s003.pdf]

S3 Table: Cross-sectional cognitive performance and hippocampal volume

Cognitive test	P value from likelihood test
Montreal Cognitive Assessment	0.003
Hopkins Verbal Learning Test immediate recall	0.06
Hopkins Verbal Learning Test delayed recall	<0.0001
Semantic fluency	0.01
Lexical fluency	0.3
Rey- Osterrieth Complex Figure copy	0.9
Rey- Osterrieth Complex Figure immediate	0.5
Rey- Osterrieth Complex Figure delay	0.3
Trail Making Test A	0.4
Trail Making Test B	0.5
Digit span forwards	0.2
Digit span backwards	0.009
Digit span sequencing	0.2
Digit coding	<0.0001

P-values result from hypothesis tests (likelihood tests) comparing regression models with and without hippocampal volume. N=511. Models were adjusted for: age, sex, FSIQ and social class. See Methods above: “Empirical justification for choice of resilience metrics: 1. Relationship between hippocampal size and cross-sectional cognitive performance”
