# Supplementary material for: Predicting cognitive resilience from midlife lifestyle and multi-modal MRI: A 30-year prospective cohort study
Source: PLoS One. 2019 Feb 19;14(2):e0211273. doi: 10.1371/journal.pone.0211273 (PMC6380585; doi:10.1371/journal.pone.0211273)
Supplement: S4 Table — Estimates are from mixed effects models (binomial regression) fitted longitudinal memory test scores over time (study Phase 11 to 12). Models were adjusted for: age, sex, FSIQ, social class, time from study baseline and hippocampal atrophy status. Estimates represent the odds of memory recall. The ‘Time x hippocampal atrophy’ interaction term tests whether there was a significant difference in memory decline over time between subjects with and without hippocampal atrophy. (PDF) [file pone.0211273.s004.pdf]

S4 Table: Longitudinal verbal memory decline

	Estimates	S.E.	95% CI	P value
Age	-0.032	0.004	-0.040 to -0.025	<0.001
Time	-0.04	0.002	-0.07 to -0.02	0.002
Sex[1]	0.23	0.004	0.13 to 0.33	<0.001
FSIQ	0.02	0.051	0.01 to 0.02	<0.001
No hippocampal atrophy[2]	-0.02	0.050	-0.11 to 0.08	0.7
Time x hippocampal atrophy	-0.04	0.017	-0.07 to -0.01	0.01

[1] Females vs. reference group males.
[2] Defined using Scheltens scale vs. reference group with hippocampal atrophy.

Estimates are from mixed effects models (binomial regression) fitted longitudinal memory test scores over time (study Phase 11 to 12). Models were adjusted for: age, sex, FSIQ, social class, time from study baseline and hippocampal atrophy status. Estimates represent the odds of memory recall. The 'Time x hippocampal atrophy' interaction term tests whether there was a significant difference in memory decline over time between subjects with and without hippocampal atrophy. **Abbreviations:** FSIQ – full-scale intelligence quotient, S.E. – standard error, CI – confidence interval.
