# Supplementary material for: Predicting cognitive resilience from midlife lifestyle and multi-modal MRI: A 30-year prospective cohort study
Source: PLoS One. 2019 Feb 19;14(2):e0211273. doi: 10.1371/journal.pone.0211273 (PMC6380585; doi:10.1371/journal.pone.0211273)
Supplement: S5 Table — (PDF) [file pone.0211273.s005.pdf]

S5 Table: Cross-sectional cognitive test data for resilient and non-resilient groups.

Cognitive test[1]	Resilient (N=184)	Non-resilient (N=133)	Group differences
	Mean (S.D.)	*Mean (S.D.)*	*Mean difference[2], 95% CI (p value)*
Montreal Cognitive Assessment	28.0 (1.5)	25.9 (2.6)	-2.09, -2.6 to -1.6 (<0.0001)
Category fluency	23.7 (5.4)	18.9 (5.1)	-4.9, -6.1 to -3.7 (<0.0001)
Lexical fluency	16.7 (4.1)	14.0 (4.5)	-2.7, -3.7 to -1.8 (<0.0001)
Trail Making Test A	27.6 (6.8)	38.2 (14.6)	10.6, 8.0 to 13.3 (<0.0001)
Trail Making Test B	56.4 (19.0)	89.8 (45.5)	33.4, 25.2 to 41.7 (<0.0001)
RCF copy	32.2 (2.6)	29.5 (4.8)	-2.7, -3.6 to -1.8 (<0.0001)
RCF immediate	17.3 (5.6)	12.2 (7.0)	-5.1, -6.5 to -3.6 (<0.0001)
RCF delay	17.1 (5.3)	11.9 (6.8)	-5.2, -6.6 to -3.8 (<0.0001)
HVLT immediate recall	29.6 (3.4)	24.2 (4.7)	-5.3, -6.3 to -4.4 (<0.0001)
HVLT delayed recall	10.3 (1.6)	7.3 (3.2)	-3.1, -3.7 to -2.5 (<0.0001)
Digit span forwards	11.6 (2.1)	10.0 (2.2)	-1.6, -2.1 to -1.1 (<0.0001)
Digit span backwards	10.4 (2.5)	8.7 (2.2)	-1.7, -2.2 to -1.1 (<0.0001)
Digit span sequencing	11.0 (1.9)	9.0 (2.2)	-2.0, -2.5 to -1.6 (<0.0001)
Digit coding	65.6 (11.5)	54.4(12.8)	-11.2, -13.9 to -8.5 (<0.0001)

[1] At time of the scan.
[2] From t test of means.

Abbreviations: RCF - Rey-Osterrieth Complex Figure, HVLT – Hopkins Verbal Learning Test, N – number, S.D. – standard deviation, CI – confidence interval.
