# Supplementary material for: Predicting cognitive resilience from midlife lifestyle and multi-modal MRI: A 30-year prospective cohort study
Source: PLoS One. 2019 Feb 19;14(2):e0211273. doi: 10.1371/journal.pone.0211273 (PMC6380585; doi:10.1371/journal.pone.0211273)
Supplement: S1 Fig — Predictions are made on the basis of mixed effects models (using N = 511 subjects) with cognitive testing (memory recall/20 words) performed before MRI at study Phase 11 and after the MRI in Phase 12, for a mean age of 75 years (at scan) and premorbid IQ of 118, according to cross-sectional hippocampal atrophy. Hippocampal atrophy is defined on the basis of the Scheltens scale (score>0). (PDF) [file pone.0211273.s006.pdf]

S1 Fig: Modelling longitudinal change in memory test scores according to hippocampal size for a typical subject

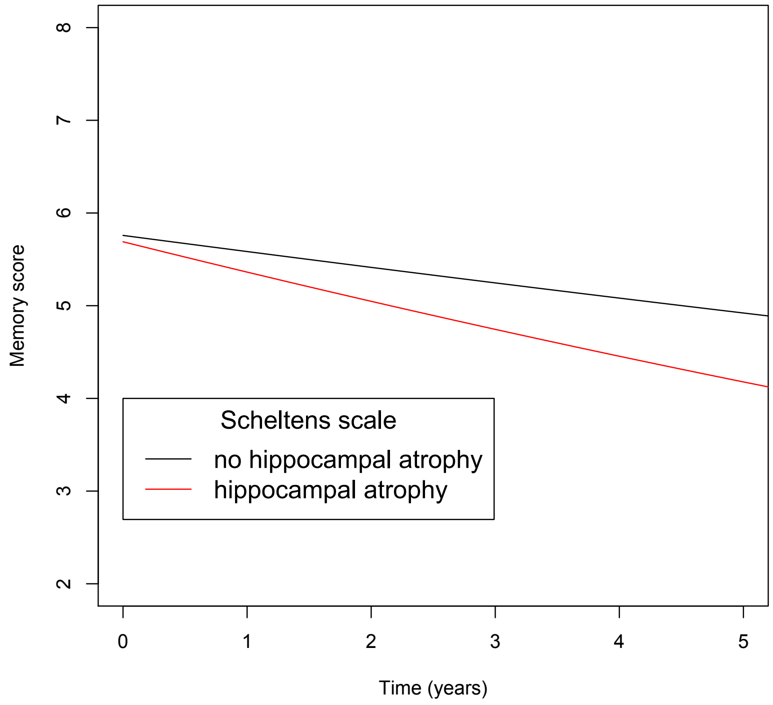

Predictions are made on the basis of mixed effects models (using N=511 subjects) with cognitive testing (memory recall/20 words) performed before MRI at study Phase 11 and after the MRI in Phase 12, for a mean age of 75 years (at scan) and premorbid IQ of 118, according to cross-sectional hippocampal atrophy. Hippocampal atrophy is defined on the basis of the Scheltens scale (score>0).
