# Supplementary material for: Predicting cognitive resilience from midlife lifestyle and multi-modal MRI: A 30-year prospective cohort study
Source: PLoS One. 2019 Feb 19;14(2):e0211273. doi: 10.1371/journal.pone.0211273 (PMC6380585; doi:10.1371/journal.pone.0211273)
Supplement: S2 Fig — Counts indicate number of participants from the whole imaged sample with hippocampal atrophy (left (L) and right (R) sides) as rated by the semi-quantitative Scheltens scale. N = 511. (PDF) [file pone.0211273.s007.pdf]

S2 Fig: Distribution of Scheltens ratings for hippocampal atrophy on whole imaged sample

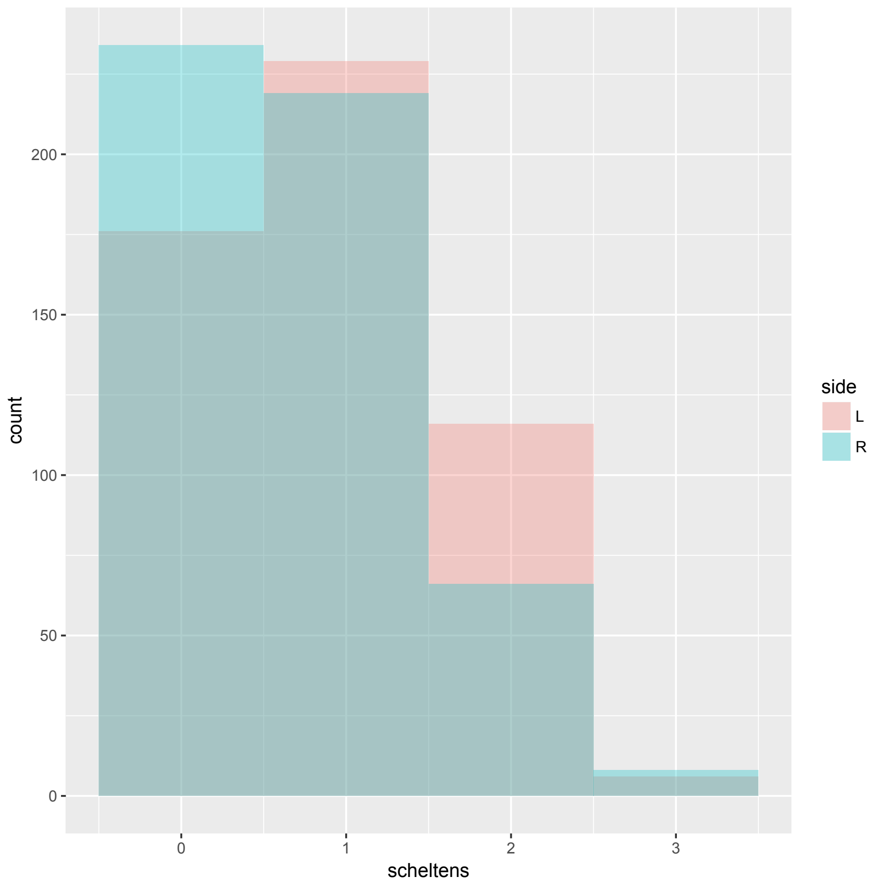

Counts indicate number of participants from the whole imaged sample with hippocampal atrophy (left (L) and right (R) sides) as rated by the semi-quantitative Scheltens scale. N=511.
