# Supplementary material for: Predicting cognitive resilience from midlife lifestyle and multi-modal MRI: A 30-year prospective cohort study
Source: PLoS One. 2019 Feb 19;14(2):e0211273. doi: 10.1371/journal.pone.0211273 (PMC6380585; doi:10.1371/journal.pone.0211273)

S3 Fig: Functional connectome consisting of hierarchical ordering of the 58 functionally distinct brain regions as network nodes, and the connectivities between these as network edges. The strengths of the estimated network edges are elements in an $N_{nodes} \times N_{nodes}$ network matrix. Boxes above the diagonal represent full correlations (thought to represent direct and indirect connections) and those below the diagonal represent partial correlations (used to infer only direct connections between nodes).

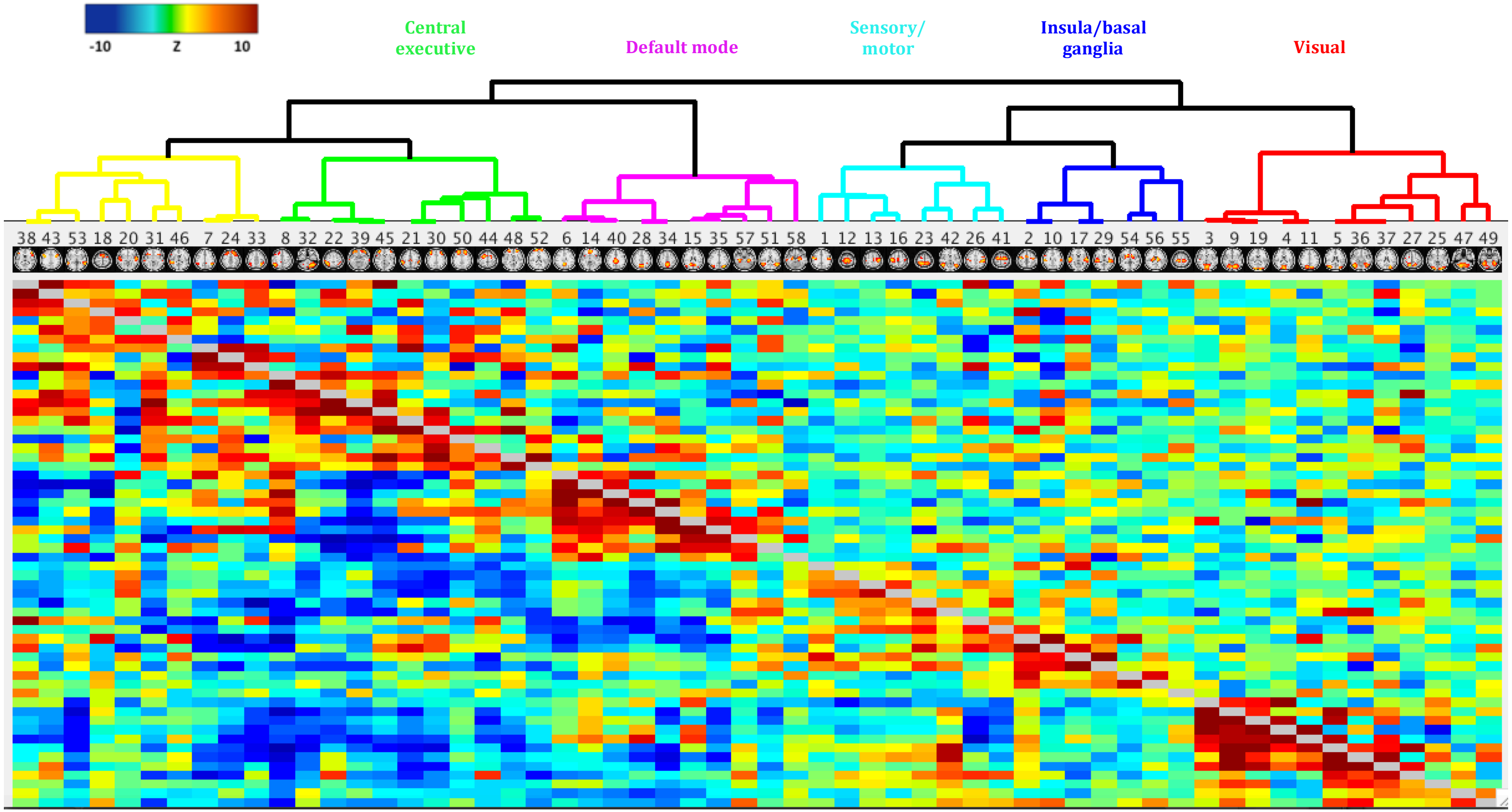

Supplement: S3 Fig — The strengths of the estimated network edges are elements in a Nnodes x Nnodes network matrix. Boxes above the diagonal signify full correlations (thought to represent direct and indirect connections) and those below the diagonal represent partial correlations (used to infer only direct connections between nodes). Based on data from 317 subjects. (PDF) [file pone.0211273.s008.pdf]
