# Supplementary material for: Predicting cognitive resilience from midlife lifestyle and multi-modal MRI: A 30-year prospective cohort study
Source: PLoS One. 2019 Feb 19;14(2):e0211273. doi: 10.1371/journal.pone.0211273 (PMC6380585; doi:10.1371/journal.pone.0211273)
Supplement: S4 Fig — Violin plots (outline includes all data points; red diamond indicates mean) display Scheltens visual rating scores against automated hippocampal (as a % of total intracranial volume) for left and right sides. Red diamonds indicate mean hippocampal volumes. N = 511. (PDF) [file pone.0211273.s009.pdf]

S4 Fig: Relationship between Scheltens ratings of hippocampal atrophy and volumetric hippocampal measures.

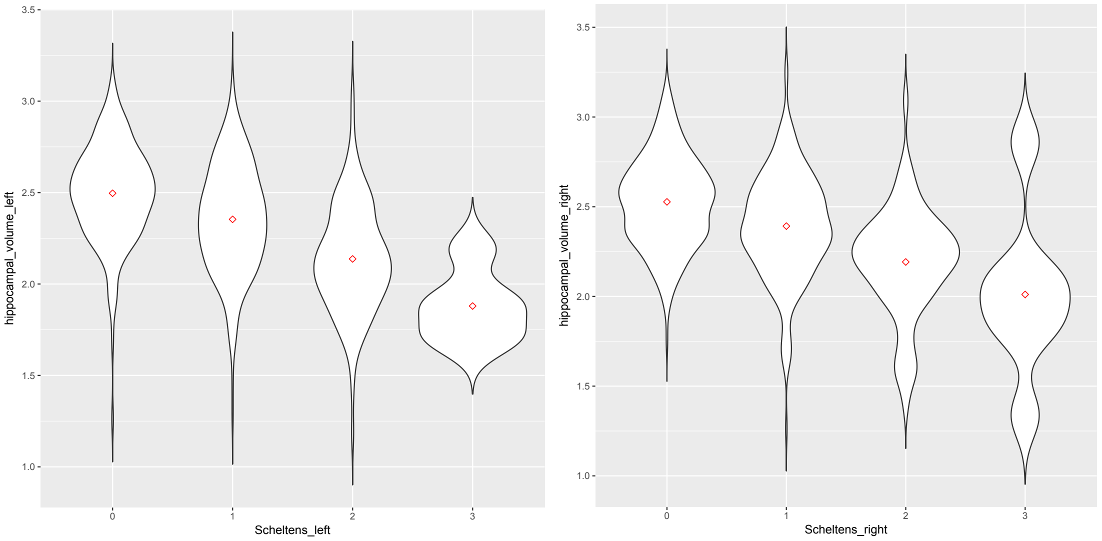

Violin plots (outline includes all data points; red diamond indicates mean) display Scheltens visual rating scores against automated hippocampal (as a % of total intracranial volume) for left and right sides. Red diamonds indicate mean hippocampal volumes. N=511.
