# Supplementary material for: Predicting cognitive resilience from midlife lifestyle and multi-modal MRI: A 30-year prospective cohort study
Source: PLoS One. 2019 Feb 19;14(2):e0211273. doi: 10.1371/journal.pone.0211273 (PMC6380585; doi:10.1371/journal.pone.0211273)
Supplement: S5 Fig — Voxel-based morphometry analysis results. Orange/red blobs represent voxels where there is significantly higher grey matter density in resilient (N = 184) compared to cognitively impaired (N = 133) groups. Colour bar indicates 1-p values. Analysis was adjusted for: age, sex, alcohol consumption, Framingham Risk Score, FSIQ, social class and TFCE-corrected. (PDF) [file pone.0211273.s010.pdf]

S5 Fig: Grey matter in resilient and non-resilient subjects

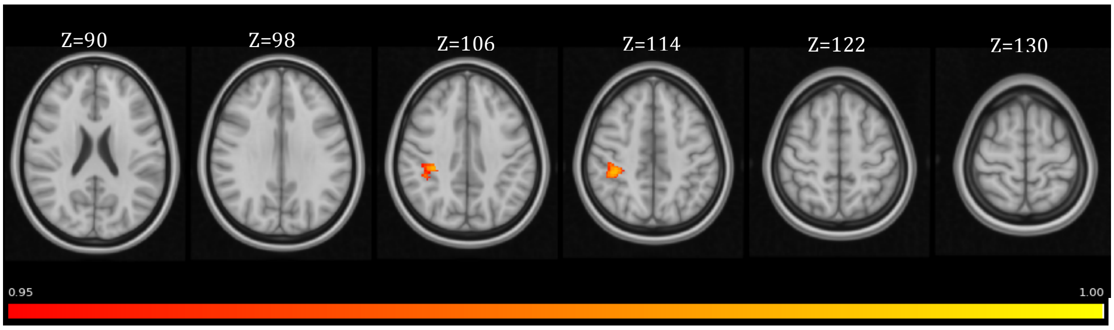

Voxel-based morphometry analysis results. Orange/red blobs represent voxels where there is significantly higher grey matter density in resilient (N=184) compared to cognitively impaired (N=133) groups. Colour bar indicates 1-p values. Analysis was adjusted for: age, sex, alcohol consumption, Framingham Risk Score, FSIQ, social class and TFCE-corrected.

Abbreviations: Z – spatial axis, FSIQ – full-scale intelligence quotient, TFCE – threshold-free cluster enhancement.
