# Supplementary material for: Predicting cognitive resilience from midlife lifestyle and multi-modal MRI: A 30-year prospective cohort study
Source: PLoS One. 2019 Feb 19;14(2):e0211273. doi: 10.1371/journal.pone.0211273 (PMC6380585; doi:10.1371/journal.pone.0211273)
Supplement: S1 Text — (PDF) [file pone.0211273.s011.pdf]

S1 Text

1. Relationship between hippocampal size and cross-sectional cognitive performance

We empirically tested which cognitive tests correlated with hippocampal size in our sample. This was achieved by fitting regression models to check whether hippocampal volume (independent variable) predicted cross-sectional performance on a range of memory tests (dependent variable). Age, sex, FSIQ and social class were included as covariates. Where the test score represented a continuous variable, multiple linear regression was used. For count data, initially Poisson regression was fitted and checked for over-dispersion. If this was found, a negative binomial model was used. For the remainder of the tests, where the upper score is bounded, regression models were initially fitted using binomial distributions. In cases of over-dispersion a folded transformation was performed and checked for approximate normality using Q-Q plots of residuals. The same models were re-fitted with and without hippocampal size, and a hypothesis test (likelihood ratio) was performed. Calculated p-values were used to test whether hippocampal size made a significant difference to the model (**S5 Table**).

2. Relationship between hippocampal size and future cognitive decline

We used mixed effects models to test whether hippocampal atrophy, as defined using the Scheltens scale, predicted future memory decline over approximately a 5-year period between Phases 11 and 12 of the Whitehall II study. Binomial regression was used in view of count data (Whitehall II verbal memory test = words recalled from a list of 20 (see Topiwala et al. 2017; **S6 Table**). In order to test whether cognitive decline significantly differed between those with and

without hippocampal atrophy, interaction terms between time and Scheltens category were added. Models were visually presented using graphs to predict trends in cognitive test scores over the study for a “typical” participant – male, of mean age (75 years), FSIQ (118 years) (**S7 Fig**).

Topiwala A, Allan CL, Valkanova V, Zsoldos E, Filippini N, Sexton C, et al. Moderate alcohol consumption as risk factor for adverse brain outcomes and cognitive decline: longitudinal cohort study. BMJ. 2017; 357: j2353.
